# In Search of the Lost Interaction: A Theoretical and Methodological Framework for Researching Interactions

**DOI:** 10.5964/ejop.14957

**Published:** 2025-08-29

**Authors:** Geoffrey Schweizer, Maximilian Köppel

**Affiliations:** 1 Department of Sport and Exercise Psychology, University of Heidelberg, Heidelberg, Germany; 2 Department of Medical Oncology, National Center for Tumor Diseases, Heidelberg University Hospital, Heidelberg, Germany; Massey University, Auckland, New Zealand

**Keywords:** replicability, positive predictive value, power, prior probability, pattern of means

## Abstract

We suggest that psychological research into interaction effects might benefit from analyzing potential interactions from the perspective of the Positive Predictive Value (PPV). The PPV denotes the post-study probability that a claimed effect is true, based on the pre-study probability that said effect exists, the power of the respective test and the significance level used for testing. We use the PPV in order to propose a framework structuring potential interaction effects based on their (theoretical) plausibility and their shape. Specifically, the position of a hypothesized interaction in the proposed framework may inform sample-size planning and the choice of alpha levels prior to a study; and it may inform confidence into results after a study. Finally, we present a heuristic approach for planning research on interactions based on R (the pre-study probability that an effect exists), the PPV (the post-study probability that a claimed effect is true) and α (the significance level used for significance testing). In doing so, we aim to provide a nuanced view on the feasibility of investigating into interactional hypotheses, a view that is critical where needed but that at the same time does not discourage research on interactions.

Recent replication studies suggest that interactions may have poorer replicability than main effects (Altmejd et al., 2019; OSC, 2015). This difference is so large that it emerges as one of the most important predictors of replicability (Altmejd et al., 2019). At the same time, several authors caution that testing at least certain kinds of interaction effects may be more difficult than researchers often assume, due to a need for large sample sizes (Gelman et al., 2021; Lakens, 2020; Simonsohn, 2014; Sommet et al., 2023). Given that interactions can be considered central for psychological research, these warnings appear to be worrisome for at least two reasons: First, because they suggest that effects that are central to psychological theorizing may be harder to find and less robust than was previously thought. Second, because they might lead to the unintended consequence that researchers avoid examining interaction effects, because they consider it to be infeasible from an economical perspective.

The goal of the present paper is to investigate potential reasons for interactions’ low replicability, both reasons already presented in the existing literature and potentially novel ones. We propose that the Positive Predictive Value (PPV) constitutes a useful framework for assessing and integrating different factors contributing to interactions’ potentially low replicability, including, but not limited to statistical power when testing for interactions. The PPV denotes the post-study probability that a claimed effect is true, based on the pre-study probability that said effect exists, the power of the respective test and the significance level used for testing (Button et al., 2013; Ioannidis, 2005; Wilson & Wixted, 2018). Furthermore, we aim to present a framework structuring potential interactions based on their (theoretical) plausibility and their shape, both of which may not only affect interactions’ replicability, but also be relevant when planning original studies and when interpreting their results. Finally, we present a heuristic approach that supports researchers in planning research on interactions based on R (the pre-study probability that an effect exists), the PPV (the post-study probability that a claimed effect is true) and α (the significance level used for significance testing). With this endeavor, we hope to provide a nuanced view on the feasibility of investigating interactional hypotheses, a view that is critical where needed but that at the same time does not discourage all research on interactions.^1^1In a highly insightful paper, Rohrer and Arslan (2021) discuss several theoretical and methodological issues with interactions. However, they focus on “reliably mistaken conclusions” (Rohrer & Arslan, 2021, p. 1). That is, in contrast to the issues discussed in the present paper, the issues discussed by Rohrer and Arslan are associated with effects that can be replicated, but that lead to the wrong conclusions regarding the research question.

## The Replicability of Interaction Effects

In 2019, Altmejd and colleagues investigated the question which study characteristics can be used in order to predict replication success via black-box statistical models. They employed data from four replication projects, namely the Reproducibility Project in Psychology (RPP; OSC, 2015), the Experimental Economics Replication Project (EERP; Camerer et al., 2016), Many Labs 1 (ML 1; Klein et al., 2014) and Many Labs 3 (ML 3; Ebersole et al., 2016), totaling 131 attempts at replicating empirical effects (for more details on the data used by Altmejd et al., 2019 see Appendix A in Schweizer & Köppel, 2025a). After training several models via machine-learning algorithms, Altmejd and colleagues identified variables predicting replication success. One of the variables most strongly predicting replication success (after *p*-values and effect sizes) is “whether central tests describe interactions between variables or (single-variable) main effects” (Altmejd et al., 2019, p. 11). In their data, “eight of 41 interaction effect studies replicated, while 48 of the 90 other studies did” (Altmejd et al., 2019, p. 11).^2^2Of these 41 interaction effects, 37 come from the RPP, 3 from ML 3, and 1 one from the SSRP (Social Sciences Replication Project; Camerer et al., 2018). Data from the SSRP were not used for setting up the model, however they were used for validating the model via out-of-sample prediction. In other words, whereas roughly half of the investigated main effects replicated, only about one fifth of interactions did.

Altmejd and colleagues (2019) provide three tentative explanations for the lower replication success of interactions. First, they point out that interactions may be “slippery statistically” (Altmejd et al., 2019, p. 11), as they are subject to measurement error in more than one variable. Second, they speculate that interactions are particularly likely to be the result of *p*-hacking. For example, when researchers do not find a hypothesized main effect in the whole sample, they may look for the respective finding in different subsamples (e.g., based on gender or personality) until they find it in one. Third, they point out that underpowered research is less likely to replicate (e.g., Fraley & Vazire, 2014) and that there is reason to believe that studies testing interactions may have on average lower power than studies testing main effects.

We are convinced that the third explanation in particular offered by Altmejd and colleagues (namely that some interactions did not replicate because the original research was underpowered and thus claimed effects were false) holds potential to explain why interactions are substantially less likely to replicate than main effects. This is because in the meantime, several authors have shown that research on interaction effects may require larger sample sizes in order to achieve sufficient power than researchers might have been aware of (Blake & Gangestad, 2020; Lakens, 2020; Sommet et al., 2023). Still, we agree with Altmejd and colleagues (2019, p. 11) when they conclude that “the replicability difference is striking and merits further study”. It is in this vein that we propose looking at the replicability of interactions from the perspective of the Positive Predictive Value (PPV). We consider the PPV to be helpful for this end as, a) it offers a perspective that unifies several influential concepts in one equation, b) can be linked to both planning studies and interpreting study results, and c) can be utilized to predict and understand the replicability of study results. Furthermore, the PPV combines theoretical and methodological considerations, thus bridging a gap between theory of science and methodology.

## The Positive Predictive Value (PPV)

The PPV is defined as the post-study probability that a claimed effect is true (Button et al., 2013; Ioannidis, 2005; Wilson & Wixted, 2018). In the context of NHST this usually means the probability that a significant effect is true. The PPV is defined according to Equation 1 (see below) and is a simple application of Bayes’ Theorem:

PPV = ([1 – β] x R) / ([1 – β] x R + α),1

*R* is the pre-study probability that an effect exists. (1 – β) is the studies’ power and α is the significance level used for significance testing. From a Bayesian perspective, the PPV equals the probability that a particular Hypothesis H is true given the statistical test reaches statistical significance (i.e., *p* < alpha) *p*(H = True | *p* < alpha); the power (1 – β) of the study equals the likelihood p(D|H = True); which is the probability to observe the data given the hypothesis is true; *R* resembles the prior probability that the hypothesis is true *p*(H = True); and finally α can be described as *p*(D|H0 = True), i.e. the probability of the data given the effect is not existent. Therefore, the denominator, [1 – β] x R + α, is the marginal probability of observing any positive test result regardless of whether it is a true or a false positive *p*(D), which is necessary in order to normalize the PPV within a range between 0 and 1.

Another way to illustrate the PPV is to consider statistical tests analogous to diagnostic tests, where the existence of an effect would resemble the existence of a disease. In this case, the PPV is the probability that a person is actually sick when they received a positive test result. Statistical power (1 – β) would be the sensitivity of the test, i.e., the probability the diagnostic identifies the disease if the tested person is indeed sick. The prior probability would equal the prevalence of the disease in the population, i.e., the unconditional probability that a random person has this particular disease; and finally, α is the probability for a false-positive, i.e., that the diagnostic identifies an apparently healthy patient as sick (in diagnostic terms 1 minus the specificity of the diagnostic). Therefore, the denominator would be the totality of positive rest results.

*R* is defined as the base rate of true effects among the population of investigated effects in a given field (Wilson & Wixted, 2018). This parameter is also called “the unconditional probability of a true effect” (Miller & Ulrich, 2016, p. 666). That is, when categorizing study outcomes as true positives, false positives, true negatives and false negatives, *R* equals the sum of the probabilities of true positives and false negatives (Miller & Ulrich, 2016).

*Power* is defined as the probability of finding an effect of a certain size or larger given that it exists, or as correctly rejecting a null hypothesis (Button et al., 2013). Power depends on effect sizes (the larger the effect size, the higher the power), sample sizes (the larger the sample, the higher the power), α (the lower alpha, the lower the power) and research designs (some research designs provide more power than others).

From the formula presented above, it follows that for a given *R* and given α the lower the power the lower the PPV (Button et al., 2013; Wilson & Wixted, 2018). Likewise, it follows that for a given power and given α the lower *R* the lower the PPV. Thus, two studies claiming an effect with the same power and the same level of significance may have different probabilities of said effects being true, given they had different *R*s. In Appendix B (see Schweizer & Köppel, 2025a) we present the results of some simulations showing how the PPV changes depending on different values of *R* for given levels of (1 – β) and α: please see Schweizer and Köppel (2025b) for the code used for the simulations. These simulations demonstrate a well-known yet consequential effect, namely that the effect of *R* on the PPV is higher when power is lower (and vice versa).

From the PPV it follows that fields with higher power and higher *R*s have more true findings than fields with lower power and lower *R*s (Wilson & Wixted, 2018). As true effects are more likely to replicate than false effects (assuming that researchers employ dependable methodology), the former fields should have higher replicability than the latter ones (Wilson & Wixted, 2018). In the next chapter we show how the PPV can be used in order to gain an improved understanding of interaction effects and their respective replicabilities. We will do so by focusing on the PPV’s different components as they relate to interaction effects. First, we will discuss power considerations and sample size requirements regarding interaction effects. Second, we will discuss considerations regarding interaction effects’ prior probabilities of being true (i.e., their *R*s). In a third step, we will combine both considerations in a single framework. Next, we will offer some suggestions for researchers planning studies involving interactional hypotheses.

## Interaction Effects From the Perspective of the PPV

### Power and Sample Size Requirements for Testing Interaction Effects

From the perspective of the PPV, power is not only defined as the probability of finding an effect of a certain size given that it exists, but it also influences a finding’s post-study probability of being true. However, what is known about power when testing for interaction effects? Recently, several authors have argued that depending on the nature of an interaction, large sample sizes may be required in order to achieve sufficient power (Blake & Gangestad, 2020; Lakens, 2020; Simonsohn, 2014; Sommet et al., 2023). Furthermore, these authors have argued that many psychologists may not be aware of the real sample-size requirements when testing for interaction effects.

**Figure 1:**
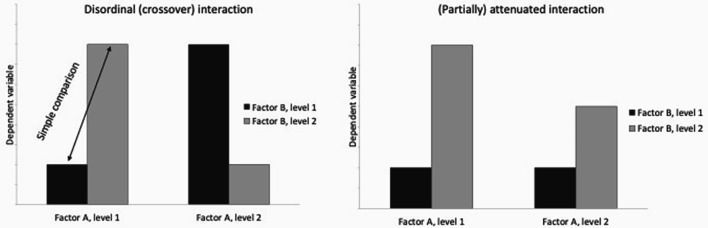
An Example of a Disordinal and an Example of a Partially Attenuated Interaction in a 2 x 2 Design

Generally, as with all kinds of effects, power, and thus sample size depends on the size of an interaction’s effect.^3^3Generally, the considerations presented in this manuscript apply to both categorical variables (e.g., an independent variable in an experimental design) and continuous variables (e.g., age as a moderator). For sake of simplification, many authors refer to categorical variables in a 2 x 2 design (e.g., Lakens, 2020; Sommet et al., 2023). Particularly when referring to their work, we adopted this practice. In order to understand sample-size requirements for testing for an interaction, it is necessary to distinguish between different kinds of interaction effects based on their shape, but also on their ‘function’ (Blake & Gangestad, 2020; Lakens, 2020; Simonsohn, 2014; Sommet et al., 2023) (Figure 1). Regarding their *shape*, one can distinguish between disordinal and ordinal interactions. A disordinal interaction (also called cross-over interaction) “occurs when the group with the larger mean switches over” (Lakens, 2020, p. 3; see also Sommet et al., 2023). In ordinal interactions, “the mean of one group is always higher than the mean of the other group” (Lakens, 2020, p. 3; see also Sommet et al., 2023). A special kind of ordinal interactions are called attenuated interactions, which by definition serve a certain *function*. “Attenuated interactions [….] characterize situations where the effect of a moderator is to reduce or eliminate, but not reverse, the main effect” (Blake & Gangestad, 2020). Although attenuated interactions are ordinal interactions when looking at their shape, there is an additional element in their definition: They are defined in relation to a main effect, that they are supposed to reduce (partially attenuated interaction) or eliminate (fully attenuated interaction).

Different authors have approached interaction effects’ power requirements from different perspectives. Sommet et al. (2023) try to arrive at the most general recommendations based on generic assumptions regarding the plausibility of different effect sizes for interactions. Sommet et al. (2023) assume that disordinal interactions typically have the same effect size as a median-sized main effect, that fully attenuated interactions typically have half the size of a median-sized main effect (and thus of a typical disordinal interaction), and that partially attenuated interactions typically have between one fourth and one fifth the size of a median-sized main effect (and thus of a typical disordinal interaction). Based on these assumptions they calculate the sample sizes required for finding interaction effects of the respective sizes.^4^4For details on these calculations and the underlying formulas please see Sommet et al. (2023). They show that (when accepting the assumptions regarding typical sizes), in order to obtain a power of .80, researchers need 256 participants for finding a typical disordinal interaction, 1024 participants for finding a typical fully attenuated interaction and 5575 participants for finding a typical partially attenuated interaction. They go on to conduct a metastudy suggesting that most psychological studies fall short of these sample sizes. One potential limitation of these conclusions is that Sommet et al. might underestimate the size of typical interactions (Sommet et al., 2023). The following two approaches circumvent this limitation by not making recommendations based on general assumptions regarding effect sizes, but by basing their recommendations on more specific comparisons.

Both Simonsohn (2014) and Blake and Gangestad (2020) avoid general assumptions regarding interactions’ effect sizes and instead compare interaction effects to main effects in the particular case of attenuated interactions. As attenuated interactions are defined in relation to a main effect, we can determine the sample size that is needed in order to find the attenuated interaction effect with the same power as the respective main effect, without making any assumptions about effect sizes of (ordinal) interactions in general. Based on both mathematical derivations and simulations, Simonsohn (2014) and Sommet et al. (2023) show that when researchers need a specific sample size for finding a certain main effect with a certain power, then they need four times that sample size for finding the respective *fully attenuated* interaction with the same power. When they are looking for a *partially attenuated* interaction, then the sample size needs to be even larger as compared to the sample size needed for finding the main effect (Simonsohn, 2014; Sommet et al., 2023). To the extent that published original effects did not follow these rather challenging sample size requirements, they were not adequately powered and are thus less likely to replicate. Blake and Gangestad (2020) provide further illustrations of attenuated interactions’ need for large sample sizes using real-world examples from published studies.

Lakens (2020) compares ordinal and disordinal interactions “where the largest simple comparison has the same effect size”. Simple comparisons refer to the effects of one independent variable separately for the levels of the second independent variable. Regarding the shape of interaction effects (or their “pattern of means”, Lakens, 2020), Lakens shows that “*in two studies where the largest simple comparison has the same effect size*, a study with a disordinal interaction has much higher power than a study with an ordinal interaction”. In a specific example, Lakens (2020) compares two hypothetical studies with the same sample sizes, however one with an ordinal and one with a disordinal interaction. In both studies, the largest simple comparison has the same size. In this example, the power for finding the disordinal interaction is nearly three times as high as the power for finding the ordinal interaction. It follows that when planning sample sizes for finding interaction effects, researchers are well advised to take into account the shape of the respective interaction.

However, it would be wrong to conclude that interactions always require large sample sizes in order to be detected with sufficient power. First, as noted above, power, and thus sample size depends on the size of the respective effect. Thus, if researchers can reasonably assume a large effect size, a correspondingly small sample size will be required. Second, as shown by Lakens (2020), effect sizes for disordinal interactions are larger than effect sizes for ordinal interactions, given a largest simple comparison of the same size. Thus, when a theory allows researchers to hypothesize a disordinal interaction, they need a smaller sample size in order to obtain a certain power than when the theory allows hypothesizing an ordinal interaction, again given a largest simple comparison of the same size.

In past publications, thinking about these issues has sometimes been obfuscated, because, a) researchers referred to interactions without specifying their size or their type, or b) because researchers explicitly or implicitly assumed that interaction effects can never be larger than main effects, or c) because researchers only referred to ordinal interactions in their considerations or examples (Lakens, 2020). For example, Gelman and colleagues (2021, p. 301) use an ordinal interaction of either the same size or half the size of a main effect when discussing why “[i]interactions are harder to find than main effects”. Likewise, Maxwell and colleagues assume that interactions are often small and ordinal (Maxwell et al., 2018). Thus, it is sometimes assumed that researchers always need large samples in order to detect interaction effects, because interaction effects must be smaller than main effects, either per se or because they are attenuating interactions. Having looked at interactions from the perspective of power and sample size planning, we will now turn to the next element of the PPV, namely their pre-study probabilities of being true.

### Interactions’ Pre-Study Probabilities of Being True (R)

Hypothesized interaction effects, like all other hypothesized effects, have different pre-study probabilities of being true (e.g., Button et al., 2013; Wilson & Wixted, 2018). In other words, they vary continuously on a dimension from zero (low *R*) to one (high *R*). Whereas *R* as a theoretical parameter has a clear definition (i.e., the base rate of true effects among all effects tested in a field), it is somewhat less clear which factors affect *R* and how researchers are supposed to estimate *R*. In a very general way, *R* depends on “established knowledge” (Wilson & Wixted, 2018, p. 191): The more prior knowledge we have in a specific field, the less false effects get subjected to empirical tests, and thus, the base rate of true effects among all effects tested in this field increases. In a more specific way, *R* is supposed to be higher to the extent that a hypothesis, a) “is guided by detailed, quantitative and well-supported theories” (Miller & Ulrich, 2016, p. 685), b) is more strongly supported by previous evidence, and c) is based on common experience (Miller & Ulrich, 2016; Wilson & Wixted, 2018).

Thus, interactions can be considered to be towards the low-*R* end of the dimension when they are, a) based on weaker theorizing, b) less strongly supported by previous research, and c) less in line with common experience. Interactions can be considered to be towards the high-*R* end of the dimension when they are, a) based on stronger theorizing, b) more strongly supported by previous research, and c) more in line with common experience. For example, when researchers find a main effect and then test for an interaction of their main effect with gender, without there being a theoretical reason to do so or prior research reporting such an interaction, their hypothesized effect would have a low prior probability of being true (i.e., it constitutes a low-*R* interaction). However, when researchers have a theoretical reason to expect an interaction effect, or when substantial prior research has reported similar effects, then their hypothesized effect would have a higher prior probability of being true (i.e., it constitutes a high-*R* interaction).

To the extent that low-*R* interactions make up a sizeable proportion of all interactions tested in a field, interaction effects in this field will on average have low PPVs and thus poor replicability. Thus, one reason for interactions’ poorer replicability may be that interactions tested in recent psychological research *on average* had lower *R*s than main effects. More precisely, we will argue that some interactions are particularly prone to having low *R*s (whereas others may have high *R*s).

### The Replicability of Interaction Effects: The Current Perspective

From the current perspective, interaction effects should have a lower replicability than main effects when, a) reported interaction effects in a field have *on average* lower *R*s than main effects in this field, when b) reported interaction effects in a field are *on average* based on studies with lower power than main effects in this field, and when c) there is a combination of the previous factors. We are convinced that there is reason to believe that all of these factors came true in past research on interaction effects: As described above, sample size requirements based on interactions’ shape have only been described recently (Lakens, 2020; Simonsohn, 2014; Sommet et al., 2023). Thus, many studies were probably strongly underpowered, even more so than they were for finding main effects (Fraley & Vazire, 2014). Furthermore, we assume that more interactions than main effects had a low pre-study probability of being true (in other words, that interaction effects on average had a lower *R* than main effects) due to common research strategies outlined below.

First, so-called ‘control variables’ (e.g., gender, age) may be included as moderators into research designs based on sparse theoretical foundations and few previous findings, making them low *R* by default. Second, researchers may first establish a main effect based on theoretical or empirical grounds and then start looking for an interaction qualifying (or moderating) that main effect. In this case, the resulting effect will be low *R* and have an attenuating shape. This tendency may have been enhanced by the ‘hype’ around moderation and mediation in at least some subfields of psychological research, where finding evidence for mediation greatly increased a paper’s chances to be published in top journals (Fiedler et al., 2018). Although mediational analysis itself does not involve testing for interactions, interactions still play a role in mediational analysis (MacKinnon et al., 2007): First, because an assumption for tests of pure mediation is that the independent variable and the mediator variable do not interact. Thus, any test for mediation should include a test for an interaction between the mediator and the independent variable (MacKinnon et al., 2007). Second, researchers may test for moderated mediation or mediated moderation, both of which again require testing for interactions (as moderation is statistically modelled as an interaction in moderator analysis) (MacKinnon et al., 2007). Furthermore, it seems safe to say that many researchers regarded interactions as more interesting and more newsworthy than main effects, again increasing the incentive for researchers to search for these interactions (see Rohrer & Arslan, 2021, for a similar point). Third, another common research strategy may have been to try and combine two (more or less established) theories in a novel way. The resulting hypotheses were often interactional in nature, and, due to their novelty, low *R*.

We would like to strongly emphasize that we do not object towards any of the above-described research strategies *per se*. Quite the contrary, some of them may be essential for progress in psychology; however, *only* if performed correctly. Thus, both when planning studies and when interpreting them, researchers should be aware of the role of interactions’ shape and interactions’ *R* and they should proceed accordingly. To the extent that this was not the case in past studies, interactions may have had both lower power and lower *R*s than main effects and thus should be less likely to replicate. For future research, we hope that researchers take the respective factors into account, starting from the choice of research strategy, continuing when planning studies and finally when interpreting their results. The following framework might be helpful for this end.

### Combining Power and R for Interaction Effects

**Figure 2:**
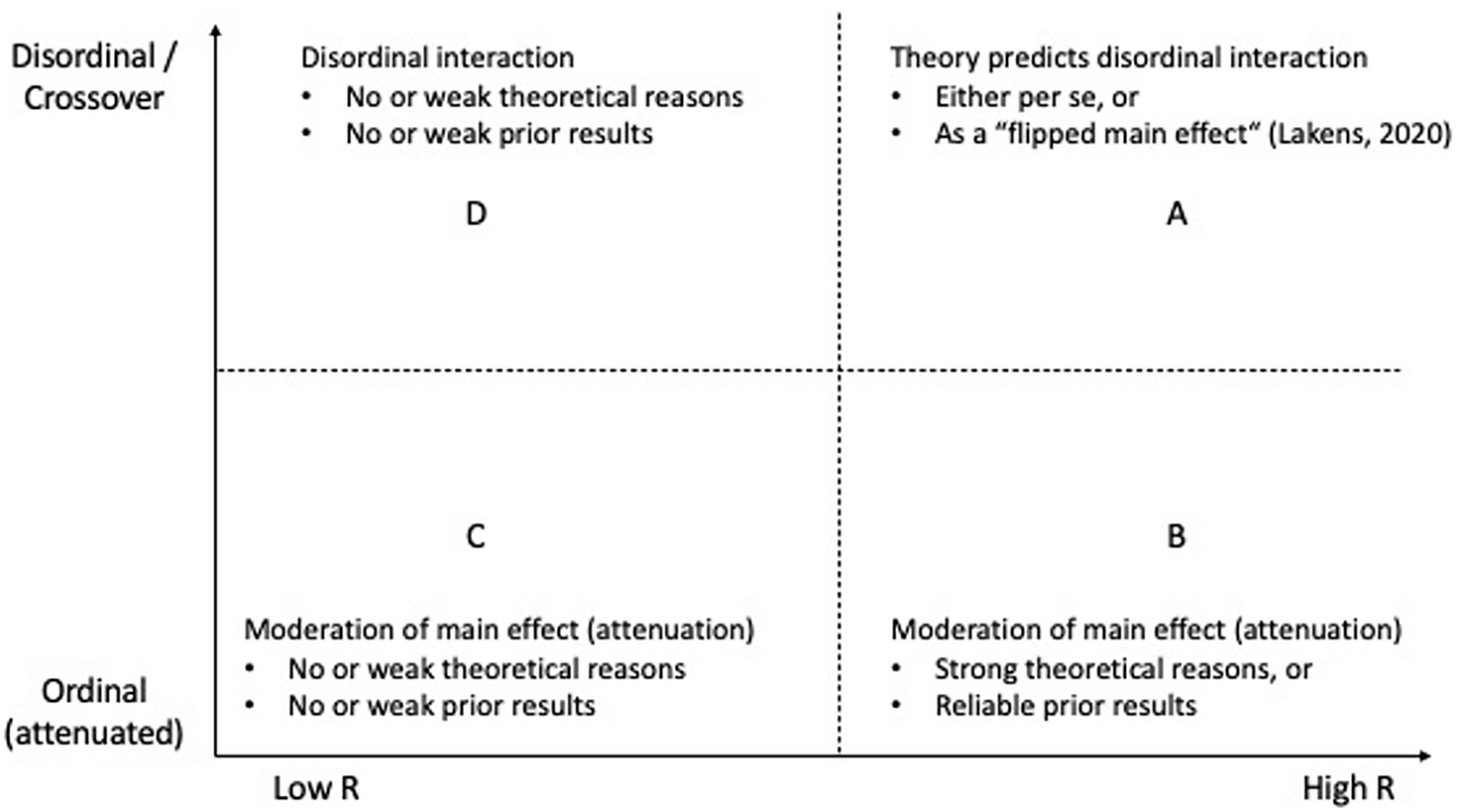
Combination of Interaction Effects’ Shape With Their *R* in a Two-Dimensional Grid *Note*. The y-axis represents the shape of interaction effects from ordinal to disordinal. The x-axis represents their prior probability of being true from low to high.

It seems possible to combine the shape of hypothesized interaction effects (with its implications for sample size planning) with their prior probability of being true, leading to a two-dimensional grid (Figure2). The y-axis represents the shape of interaction effects and depicts a continuum from ordinal to disordinal (or crossover). The x-axis represents the prior probability of being true of interaction effects and depicts a continuum from low-*R* to high-*R*.

Sector A appears to combine the best of both worlds: A high prior probability of being true with a shape that requires comparably small sample sizes in order to achieve sufficient power. Researchers who can predict a disordinal interaction on theoretical grounds can thus hope to find an interaction effect that has a high likelihood of being true and thus a high likelihood of being replicable based on comparably small sample sizes. Consequentially, it is precisely these kinds of interactions that readers should place most trust into.

Sector B contains potential interaction effects that appear to be important for theoretical and practical reasons, however that are hard to detect statistically: Both from a theoretical and an applied perspective it might be important to know whether a main effect is attenuated (e.g., when an intervention works better for women than for men). Thus, research on these effects seems worthwhile to conduct. However, researchers are well advised to utilize appropriate sample sizes and appropriate sample size planning. Sample size planning should be based on expected patterns of means (Lakens, 2020) and the exact nature of the expected attenuation (Simonsohn, 2014; see also Blake & Gangestad, 2020, for research on attenuated interactions). For this end, it might be helpful to use a software that allows entering hypothesized effects directly via their means (e.g., Superpower; Lakens & Caldwell, 2021; see Lakens & Caldwell, 2021, for more suggestions) or that allows drawing the shape of the expected interaction (INTxPower; Sommet et al., 2023). Depending on underlying effect sizes and the exact nature of the assumed attenuation, sample sizes may need to be very large in order to achieve appropriate power. Attenuated interaction effects found in small sample studies should be treated with care.

Sector C can be considered the ‘danger zone’ of research on interaction effects, as it combines the worst of both worlds: A low prior probability of being true with a shape that requires large sample sizes in order to achieve sufficient power. In fact, depending on underlying effect sizes and the exact nature of the assumed attenuation, these requirements may not only be challenging but sometimes hard to meet (Gelman et al., 2021; Simonsohn, 2014). Interaction effects fall in this sector when researchers test for attenuated moderation without having strong theoretical reason to do so. An example for this might be ordinal interactions with gender, that arise as the result of including gender as a control variable. If researchers insist on testing for such interactions, they are well advised to base their conclusions on sufficiently large sample sizes, taking both low *R* and the interaction’s shape into account. Again, it might be helpful to use a software that allows entering hypothesized effects directly via their means (Lakens & Caldwell, 2021; Sommet et al., 2023). Furthermore, when interpreting such interactions both authors and readers of the literature are well advised to consider that these interactions have a higher likelihood of being false positive and a lower likelihood of replicating, particularly when they are based on small sample sizes. Thus, neither theoretical conclusions, nor consequential real-world decisions nor the allocation of future research resources should easily be based on them.

Sector D contains potential interaction effects that seem to be less problematic than the ones in Sector C, however more problematic than the ones in Sector A. Although these effects also have a comparably low prior probability of being true, they are less challenging from a statistical perspective as they require smaller sample sizes due to their disordinal shape. Still, researchers might be well advised to treat them with care after they have been reported.

As sample sizes in order to investigate predictions from Sectors C and D can quickly need to be very large, researchers might want to ask themselves whether the potential benefits are worth the costs (e.g., Miller & Ulrich, 2016). For example, if investigating one potential interaction effect from Sector C requires a number of participants that would suffice for investigating several hypotheses from Sectors A or B, researchers need to decide whether the potential increase in knowledge regarding the effect is worth the effort.

### General Recommendations for Risky Predictions

Lakens (2020) cautions researchers not to shy away from “risky predictions”, just because these seem effortful to investigate. We fully agree. In the present framework, risky predictions can be found in Sectors C and D (i.e., risky predictions are predictions with a low prior probability of being true). The present framework allows deriving some recommendations on how to investigate risky predictions (see also Wilson & Wixted, 2018, for recommendations on low-*R* research; and Blake & Gangestad, 2020, for research on attenuated interactions). Generally, for given *R*s, PPVs can be improved by increasing power and by lowering alpha. Thus, when investigating low-*R* hypotheses, researchers can, a) increase power by increasing sample sizes (while holding alpha constant), b) increase power by utilizing suitable experimental designs (e.g., within-participants designs have higher power than between-participants designs), *if possible*, c) increase power by increasing the reliability of the dependent variable^5^5Whereas most of the literature on improving power in psychological research focusses on sample sizes, improving reliability seems to be a bit overlooked, potentially because researchers assume that their variables are reliable anyway. However, if there is room for improving reliability, the effects on power are substantial (see Sommet et al., 2023, for an illustration). (Sommet et al., 2023), and d) use lower alpha levels (while holding power constant) (see also Benjamin et al., 2018, for the benefits of lowering alpha levels). These recommendations beg the question: Increase as compared to what, or lower to what extent? Unfortunately, no accepted guidelines exist as of yet. However, one possibility would be to define a desired PPV for an assumed *R* and then to solve Equation 1 for power and alpha. This, of course, requires a precise assumption regarding *R*. Miller and Ulrich (2016, p. 685) discuss different methods of estimating *R*. These methods range from “researchers’ guesses” over “record keeping, and discussion with colleagues” to analyzing available evidence with specific statistical techniques (e.g., *p*-curves, Simonsohn et al., 2014). In addition to these general considerations, in the next paragraph we offer a heuristic approach for approximating the strength of *R* if direct evidence is unavailable, i.e., when studies have not yet tested the hypothesis in question.

### A Heuristic Approach for Planning Interaction Research Based on R and Power

**Figure 3:**
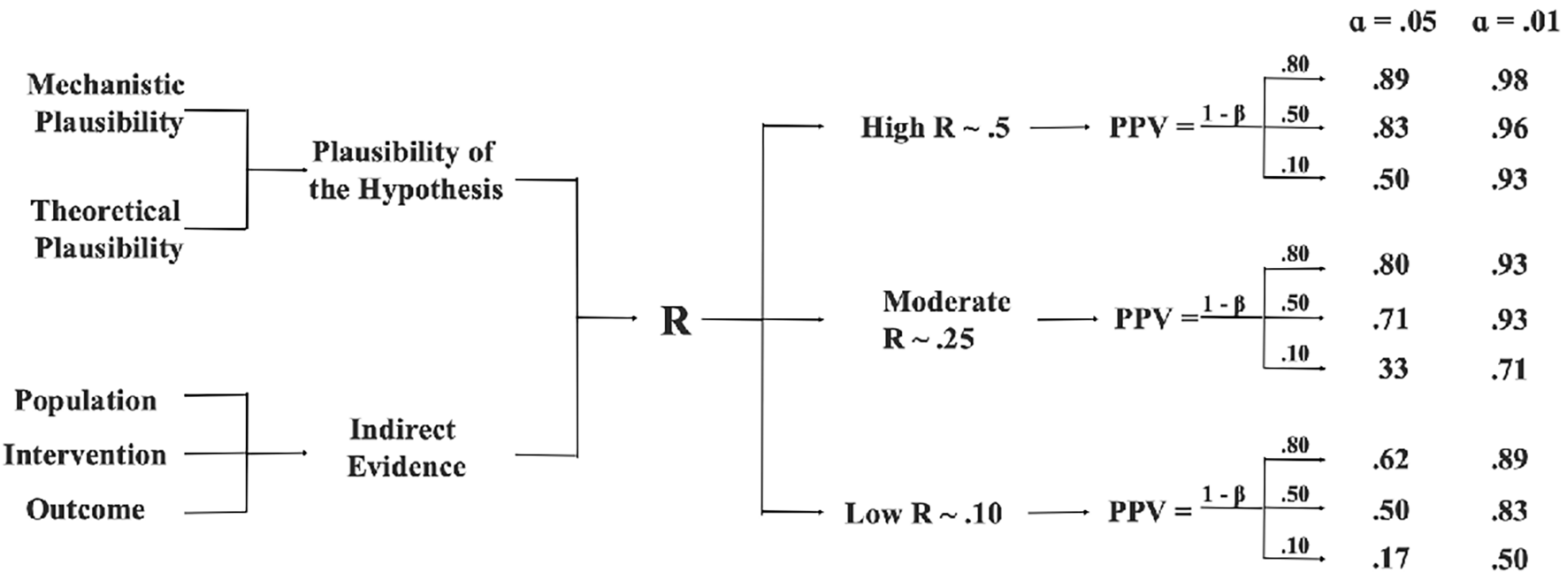
A Heuristic Approach for Planning Research Based on *R*, the PPV and α

Researchers interested in planning their research project based on the PPV, *R*, power and α can use the algorithm depicted in Figure 3 as an orientation. The left-hand side of Figure 3 presents a heuristic approach for approximating the strength of *R*. The right-hand side of the figure shows what levels of power and α are needed in order to obtain a specific PPV, based on the approximations of R made on the left-hand side. Thus, instead of solving Equation 1 for individual values of *R* and power, Figure 3 presents a simplification for selected levels of *R* and power. Figure 3 shows the PPV for different combinations of *R*, power (1 – β) and α. We combine three values of *R* (.5, .25, and .1) with three values of power (.1, .5, and .8) and two values of α (.05 and .01). The impact of these estimates on the PPV is shown on the right-hand side of the flow chart. For example, for hypotheses with low *R*, a statistically significant result — even from high-powered studies — still leaves considerable uncertainty about the truth of the result. When α is lowered to .01, the PPV increases substantially, and particularly so for either low-power or low-*R* research, or for combinations thereof.

However, low levels of power mean that researchers only have a small chance of finding an effect given that it exists, even when the corresponding PPVs can become large. For example, even when the combination of *R* = .25, power = .5 and α = .01 means that a significant result represents a true effect in 93% of cases, still only 50% of true effects become significant in the first place.

In this approach, R is influenced by two key factors (left-hand side of Figure 3): (1) the plausibility of the hypothesis, and (2) indirect supporting evidence. Plausibility can be further divided into mechanistic plausibility and theoretical plausibility.

*Mechanistic plausibility* assesses whether we know of a mechanism supporting the hypothesis. For example, in medical research, a physiological mechanism may be established in mice, before respective hypotheses are tested in humans. Likewise, in psychological research, a hypothesis that is based on an established process may seem more plausible than a hypothesis which is not based on an established process. *Theoretical plausibility* assesses whether a hypothesis is aligned with a well-established theoretical framework. For example, in line with the Theory of Planned Behavior, it seems more plausible that subjective norms have a positive association with intentions than that they have a negative association with intentions (Ajzen, 1991). Plausibility is related to the definition of *R* as the base rate of true effects tested in a field, because a field that tests more plausible effects will on average have a higher proportion of true effects than a field that tests less plausible effects.

Besides the plausibility of the hypothesis, assessing the extent of indirect evidence in support of the hypothesis can serve to inform estimates of *R*. Indirectness is a well-established concept in evidence-based medicine (EBM) (Guyatt et al., 2011). Indirectness can refer to *Indirectness of Population* (e.g., when gender moderates the effectiveness of a psychological intervention in a specific population, it is plausible to expect a respective interaction in a related population), *Indirectness of Intervention* (e.g., when gender moderates the effectiveness of a specific intervention, it is plausible to expect a respective interaction for a related intervention), and *Indirectness of Outcome* (e.g., when gender moderates the effectiveness of a specific intervention with regard to a specific dependent variable, it is plausible to expect a respective interaction with regard to a related dependent variable).^6^6In EBM, also the Indirectness of Comparator can be assessed. However, it seems difficult to find a psychological equivalent. Again, the relation to the definition of *R* is that a field that tests effects that are more strongly supported by indirect evidence will on average have a higher proportion of true effects than a field that tests effects that are less supported by indirect evidence.

Researchers interested in planning their research project based on the PPV, *R*, power and α can use the algorithm in Figure 3 as follows: First, they need to arrive at an estimate of *R* based on the mechanistic and theoretical plausibility of their hypothesis and indirect evidence in support of their hypothesis. We suggest adopting some generic levels of *R* depending on the plausibility and the availability of indirect evidence: When a hypothesis seems implausible and there is no indirect evidence, we suggest adopting a generic *R* of .10. When a hypothesis is either plausible, or there is indirect evidence, we suggest adopting a generic *R* of .25. When there is both plausibility and indirect evidence, we suggest adopting a generic *R* of .5 Of course, these values do not represent the “real *R*s” of the respective hypothesis, but they offer some orientation.

Second, researchers need to decide what level of power they want to achieve, both for optimizing power itself and with regard to the PPV. For this decision, it is helpful to consider what kind of interaction they expect (disordinal, ordinal, fully attenuated or partially attenuated), what kind of design they aim to realize (within- or between-participants), and the reliability of the dependent variable. Then, they can calculate an according sample size. When the necessary sample size seems too large, researchers can try to switch from a between-persons to a within-persons design or they can try to improve the dependent variable’s reliability. Third, they need to decide for an a priori α level in order to arrive at a PPV that they consider appropriate. The algorithm can also be used in reverse: When researchers read a paper reporting a significant result, they can try to estimate the relevant parameters and then they can decide how much trust to put into the result.

Finally, researchers might take into account the recommendations made by Rohrer and Arslan (2021), who show that researching interactions may often require more specific research questions than researchers may be aware of.

## Potential Objections, Potential Misunderstandings, Limitations and Unintended Consequences

As we have tried to make clear throughout the manuscript, we do not intend to discourage researchers from researching into interactions, from investigating risky predictions and from testing novel hypotheses. Quite the contrary, we hope to contribute a framework that is able to support and structure researchers’ work in these areas; and we hope to correct an overly pessimistic and somewhat diffuse assessment of research into interaction effects (e.g., Gelman et al., 2021). Interactions are at the heart of psychological theorizing, research and application, not the least because behavior has been fundamentally understood as being an interaction between person and situation (Furr & Funder, 2021; Lewin, 1951). Consequentially, the very idea that psychologists should refrain from research on interactions seems nonsensical and potentially disastrous to us. Thus, we agree with Rohrer and Arslan when they write “instead of putting interaction research on hiatus, we should strive for improved interaction research” (Rohrer & Arslan, 2021, p. 14). In the remainder of the manuscript, we aim to anticipate potential objections towards the current approach and to discuss some limitations.

Some authors have criticized the discussion on methodological practices in psychology in general, and the discussion on psychology’s replication rates in particular, for overly focusing on false-positive findings, while neglecting the costs of false-negative ones (Fiedler et al., 2012). We would like to point out that the present considerations address both concerns. First, high power is a remedy against both false negatives and false positives. That is, fields with higher power have on average less false-positive and less false-negative effects than fields with lower power (Button et al., 2013; Wilson & Wixted, 2018). Furthermore, considering assumed effects’ *R* can be helpful for avoiding false negatives and false positives (Button et al., 2013; Wilson & Wixted, 2018).

Even more generally, some authors have criticized the discussion on methodological practices in psychology for neglecting theoretical aspects (e.g., Fiedler, 2018). In this context, we would like to emphasize that we do not perceive the present article to be primarily or even purely methodological in nature. Instead, with this article we attempt to demonstrate how theory and methodology are inextricably linked when researching into interactions. For example, it is impossible to plan a sample size based on a hypothesized interaction’s shape, when there is no theory suitable for predicting said shape. Likewise, only properly formulated theories allow for considering an effect’s *R*. In both these examples, theoretical considerations are actually superordinate to methodological ones (Fiedler et al., 2018): First researchers need to think about their theory, then they can plan their study. However, only high-quality theories can be useful for making reliable predictions and deriving diagnostic hypotheses about an interaction’s shape or for informing *R*s (Fiedler, 2017). Thus, theory construction and formulation are of utmost importance in the present context (again, see Rohrer & Arslan, 2021, for a similar point). Indeed, we agree with Fiedler (2017, p. 1) that only a “theory-driven cumulative science” can hope to successfully solve the apparent dilemma of how to deal with potentially important, but low-*R* research. With the present paper, we aim to contribute to this endeavor.

Furthermore, although the present paper mostly refers to confirmatory research, we do not aim to devalue exploratory research. Instead, we believe that exploratory research is highly relevant for progress in psychological research, and that it should play a more prominent role (Fiedler, 2017). Indeed, it seems possible to argue that the alleged crisis in psychological research is partly due to a disregard for exploratory research in psychology, forcing researchers who engaged in valuable exploratory research to disguise it as being confirmatory. Thus, clearly separating exploratory and confirmatory research while appreciating both seems to be crucial.

In the present paper, we sometimes oversimplify matters in order to facilitate the presentation of our arguments and thus make the manuscript more accessible. For example, in some instances, we employ a binary reasoning instead of the proper continuous one. Thus, we refer to *R* and power as being low or high, although both *R* and power vary continuously from 0 to 1. Consequentially, of course, the proposed grid does not represent four kinds of qualitatively different effects. Instead, it represents a simplification of the underlying continuous reasoning. The same applies to the nature of interactions. In order to simplify matters, recent papers on interactions often refer to categorical predictors in a 2 x 2 design (e.g., Lakens, 2020; Sommet et al., 2023). Accordingly, software for calculating sample sizes for interaction research may also be limited to categorical predictors in a 2 x 2 design. Future work might try to address these limitations by addressing more complex designs and continuous moderators.

Throughout the present paper, we refer to effects as either existing or not, and to findings being false-positive or false-negative. We are aware that some authors reject the concepts of false-positive and false-negative findings or Type-1 and Type-2 errors because they invite researchers to think about theories, hypotheses and effects in a binary manner (e.g., an effect is true or a hypothesis is rejected), although effects vary and the evidence for effects is continuous (e.g., Gelman, 2013). From this perspective, instead of determining whether an effect exists or not, it makes more sense to try and estimate the probabilities of varying sizes of an effect depending on different values of additional parameters (Gelman, 2013; Gelman et al., 2012). This does not render reflections about sample sizes and prior probabilities (in this case of different sizes of effects) obsolete, quite the contrary. For example, in the context of Bayesian modelling, choosing and justifying prior distributions of potential effect sizes are crucial steps (van de Schoot et al., 2021).

Indeed, this might be a substantial objection towards the present manuscript: Why retain the paradigm of NHST instead of abandoning it and turning to Bayesian modelling entirely? While some may consider this to be the most fruitful option for psychological research (e.g., Wagenmakers et al., 2018), we do not consider it likely that psychologists will abandon NHST entirely any time soon. Thus, just like other authors (e.g., Benjamin et al., 2018; Lakens, 2021), we consider it worthwhile to try and contribute to the methodological approach that will probably be around for quite some time in the future. However, thinking about the pre-study probabilities of their effects being true might gently introduce psychologists to a more Bayesian way of thinking without them having to make a ‘hard transition’ from frequentist to Bayesian statistics in the first place. We hope that in the future, researchers in psychology will use the presented framework in order to combine theoretical and statistical considerations when conducting or interpreting research on interactions. This will hopefully contribute to high-quality research on interactions, a line of research that we consider to be essential in order to develop robust knowledge in both basic and applied psychology.

## Supplementary Materials

**Table:** 

Type of supplementary materials	Availability/Access
Code	
Annotated R code for all simulations.	Schweizer & Köppel (2025b)
Material	
a. Appendix A: Replication projects’ inclusion of interactional hypotheses.	Schweizer & Köppel (2025a)
b. Appendix B: Simulations.	Schweizer & Köppel (2025a)
